# Antioxidant Bioactivity of Extracts from Beach Cast Leaves of *Posidonia oceanica* (L.) Delile

**DOI:** 10.3390/md19100560

**Published:** 2021-09-30

**Authors:** Concetta Maria Messina, Rosaria Arena, Simona Manuguerra, Yann Pericot, Eleonora Curcuraci, Fanny Kerninon, Giuseppe Renda, Claire Hellio, Andrea Santulli

**Affiliations:** 1 Laboratorio di Biochimica Marina ed Ecotossicologia, Dipartimento di Scienze Della Terra e del Mare DiSTeM, Università Degli Studi di Palermo, Via G. Barlotta 4, 91100 Trapani, Italy; rosaria.arena@unipa.it (R.A.); simona.manuguerra@unipa.it (S.M.); eleonora.curcuraci@unipa.it (E.C.); andrea.santulli@unipa.it (A.S.); 2 LEMAR, IRD, CNRS, Ifremer, Université de Brest, F-29280 Plouzane, France; yann.pericot@outlook.fr (Y.P.); fanny.kerninon@univ-brest.fr (F.K.); claire.hellio@univ-brest.fr (C.H.); 3 Istituto di Biologia Marina, Consorzio Universitario della Provincia di Trapani, Via G. Barlotta 4, 91100 Trapani, Italy; giuseppe.renda02@unipa.it

**Keywords:** *Posidonia oceanica*, polyphenols, antioxidants, oxidative stress, photo protection

## Abstract

The marine environment is a generous source of biologically active compounds useful for human health. In 50 years, about 25,000 bioactive marine compounds have been identified, with an increase of 5% per year. Peculiar feature of algae and plants is the production of secondary metabolites, such as polyphenols, synthesized as a form of adaptation to environmental stress. *Posidonia oceanica* is a Mediterranean endemic and dominant seagrass and represents a biologically, ecologically and geologically important marine ecosystem. Within this study, methanolic and ethanolic extracts were generated from fresh and dried *Posidonia* *oceanica* leaves, with the aim to employ and valorize the beach cast leaves. The best yield and antioxidant activity (polyphenols content equal to 19.712 ± 0.496 mg GAE/g and DPPH IC50 of 0.090 µg/µL.) were recorded in 70% ethanol extracts (Gd-E4) obtained from leaves dried for two days at 60 °C and ground four times. HPLC analyses revealed the presence of polyphenols compounds (the most abundant of which was chicoric acid) with antioxidant and beneficial properties. Bioactive properties of the Gd-E4 extracts were evaluated in vitro using fibroblast cells line (HS-68), subjected to UV induced oxidative stress. Pre-treatment of cells with Gd-E4 extracts led to significant protection against oxidative stress and mortality associated with UV exposure, thus highlighting the beneficial properties of antioxidants compounds produced by these marine plants against photo damage, free radicals and associated negative cellular effects. Beach cast leaves selection, processing and extraction procedures, and the in vitro assay results suggested the potentiality of a sustainable approach for the biotechnological exploitation of this resource and could serve a model for other marine resources.

## 1. Introduction

The marine environment is characterized by a high biodiversity. Many marine organisms have developed chemical defences to adapt to the environment, producing a wide range of biologically important compounds, that can be exploited for biomedicine, food, nutraceutical and cosmeceutical purposes [1,2]. Research in the last few years has focused on algae and plants with exceptional properties [2,3,4,5,6,7]. With an estimated coverage area of over 300,000 km^2^ [8], seagrasses beds are one of the most widespread marine habitats on the planet. Despite their abundance and the fact that they offer incredible opportunities for discovering new phytochemicals, research applied to seagrasses compared to algae remains underdeveloped [9]. In particular, seagrasses have excellent human health properties, including antioxidant and anti-inflammatory activities [1,10,11,12,13]. *Posidonia oceanica* (Linnaeus) Delile *(Phylum magnoliophyta; Class liliopsida; Order alismatale*) is a seagrass endemic to the Mediterranean Sea, with an important ecological role [14]. It is protected through the EU European ‘Habitats’ Directive 92/43/EEC [15], the Bern (1976) and Barcelona Convention (1976, amended in 1995) [14,16].

In autumn *P*. *oceanica* loses leaves are deposited along the coast by storm surges and subsequently form dead leaf structures called “banquettes”, that exert a major role in coastal biodiversity end shore protection from erosion [14]. Since prehistoric times, dead leaves of *P. oceanica* have been used throughout the Mediterranean as thermal insulation, compost, etc., until the species was declared protected, leading many countries to ban its use [7,14]. In Italy, the legislation concerning the management of beached *P. oceanica* is still lacking and the matter is regulated either as a waste or as a resource [17], allowing the use of *P. oceanica* beach cast leaves as compost up to a maximum of 20% their weight in the banquettes [17,18,19]. 

In recent years, a growing number of studies have increased the knowledge of the beneficial properties of phytocomplex extracted from *P. oceanica* for human health, such as antidiabetic, antioxidant and vasoprotective effects [1,3,20]. Different natural products have been isolated from leaves, such as antioxidant molecules (e.g., natural phenols, phenylmethane derivatives, phenyl ethane derivatives, phenylpropane derivatives and their esters, chalkones, flavonols) [7,21,22]. 

The high importance of antioxidants in the prevention of the diseases, determined by oxidative stress, has drawn the attention to the natural molecules extracted from marine plants, algae and bacteria able to counteract oxidative stress caused by exogenous and endogenous factors [1,2,23,24,25,26,27,28,29]. Furthermore, the interest in identification of cosmeceutical compounds extracted by marine plants and algae is increasing [2,3,4,5,6,7]. 

*P. oceanica* leaves have a high content of secondary metabolites, mainly represented by phenolic compounds making this seagrass a potential useful source of antioxidant molecules, to counter oxidative stress, which leads wrinkle formation, skin aging, cancer and cardiovascular diseases [1,7,30,31,32,33]. Excessive and prolonged exposure to ultra-violet radiation (UV) is one of the main factor for the cellular over-production of radical species, such as reactive oxygen species (ROS) [34,35]. Under normal physiological conditions, cellular systems are able to maintain a balance between the production of reactive oxygen species and the cellular ability to detoxify the reactive intermediates [7]. 

Considering the bioactive properties of *P. oceanica* leaves extracts and its ability to counteract oxidative stress, the aims of this work were the quantification of the polyphenolic content and antioxidant capacity of extracts obtained from leaves of *P. oceanica* and the assessment of the efficiency of different solvents for polyphenol extraction. We investigated also whether the different leaf colors, indicative of the reduction of photosynthetic capacity during aging, could be linked to antioxidant capacity, correlated with a different quantity and quality of specific compounds.

In order to consider biotechnology applications, we checked whether the antioxidant power of the extract was transferred to the cellular level, by testing the bioactive properties in vitro, in terms of antioxidants and photo-protective, for application in cosmeceutical. Furthermore, we proposed a sustainable protocol for the collection and use of *P. oceanica* beach cast leaves before they die and became litter and accumulating in banquettes.

## 2. Results

### 2.1. Characterization of the Antioxidant Power

#### 2.1.1. Total Phenolic Content

**Figure 1:**
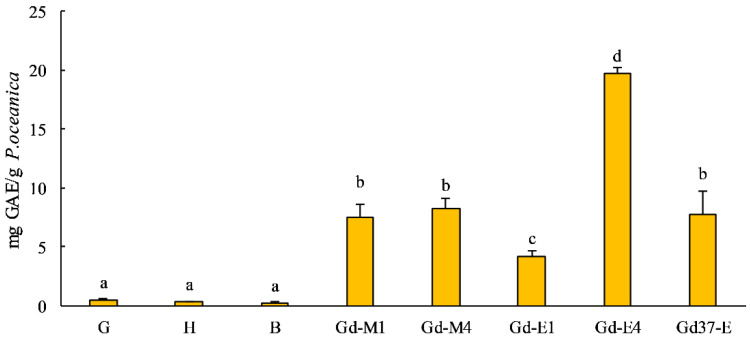
Total phenolic content (mg GAE/g *P. oceanica*) of extracts obtained from fresh and dried leaves of *P. oceanica*. G: green fresh leaf ethanol 70% extracts; H: half-green fresh leaf ethanol 70% extracts; B: brown fresh leaf ethanol 70% extracts; Gd-M1: methanol 80% extracts from green leaves dried at 60 °C and ground once; Gd-M4: methanol 80% extracts from green leaves dried at 60 °C and ground four times; Gd-E1: ethanol 70% extracts from green leaves dried at 60 °C and ground once; Gd-E4: ethanol 70% extracts from green leaves dried at 60 °C and ground four times; Gd37-E: ethanol 70% extracts from green leaves dried at 37 °C and ground four times. Results are given as means ± SD. Lowercase letters indicate significant differences between different extract (*p* < 0.05).

Figure 1 showed the phenolic yield of the various extracts obtained from *P. oceanica* leaves. 

The total phenolic contents of extracts obtained from fresh leaves by 70% ethanol classified as green (G), half-green (H) and brown (B), showed similar values. Among extracts obtained from green leaves, the higher content (*p* < 0.05) in total phenolic compounds (19.712 ± 0.496 mg GAE/g) was measured in those obtained from green leaves dried at 60 °C for 48 h, ground four times and extracted with 70% ethanol (Gd-E4).

#### 2.1.2. HPLC Analysis of Phenolic Compounds

Table 1 showed phenolic compound concentrations (µg/g) of extracts from *P. oceanica* leaves.

Extracts obtained from green leaves (G) showed the highest total concentration of phenolic compounds compared to H and B extracts (Table 1).

Extracts obtained from dried leaves (Table 1) had higher concentration (µg/g) of phenolic compounds, compared to extracts from fresh leaves. 

Solvents utilized for the extraction induced significant differences in extraction yields (Table 1). With 80% methanol extraction, both extracts obtained from dried green leaves ground once ((Gd-M1) and four times (Gd-M4), ρ-hydroxybenzoic acid is not present, while in the ethanol extracts (Gd-E1) it is present at low concentration (2.714 µg/g) and high concentration in Gd-E4 (519.272 µg/g). Chicoric acid was the most abundant phenolic compound in all extracts (Table 1).

Significant differences were recorded between the two different drying procedures (Gd37-E and Gd-E4) (Table 1). In particular, it was observed that Gd37-E contained 110.807 µg/g DW of ρ-hydroxybenzoic and 2843.772 µg/g DW of chicoric acid, whereas Gd-E4 extracts contained 519.272 µg/g DW and 4991.813 µg/g DW of ρ-hydroxybenzoic and of chicoric acid, respectively (Table 1).

#### 2.1.3. Antioxidant Power—DPPH Assay

**Figure 2:**
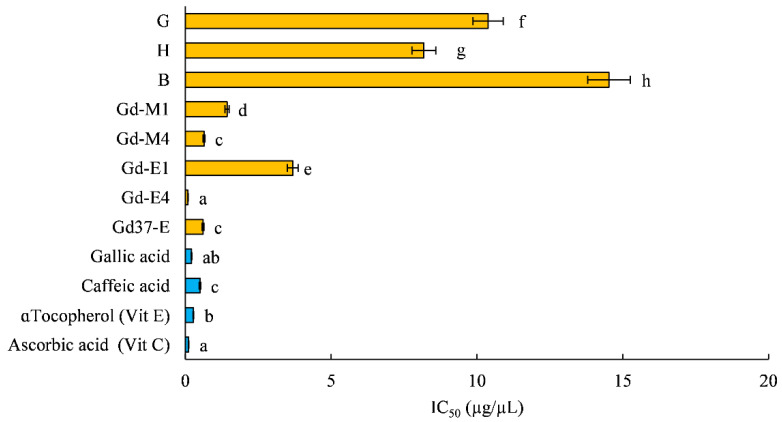
IC50 (µg/µL) of extracts of leaves of *P. oceanica* (in yellow) and IC 50 of four standards of the antioxidant power (in blue). G: green fresh leaf ethanol 70% extracts; H: half-green fresh leaf ethanol 70% extracts; B: brown fresh leaf ethanol 70% extracts; Gd-M1: methanol 80% extracts from green leaves dried at 60 °C and ground once; Gd-M4: methanol 80% extracts from green leaves dried at 60 °C and ground four times; Gd-E1: ethanol 70% extracts from green leaves dried at 60 °C and ground once; Gd-E4: ethanol 70% extracts from green leaves dried at 60 °C and ground four times; Gd37-E: ethanol 70% extracts from green leaves dried at 37 °C and ground four times. Results are given as means ± SD. Lowercase letters indicate significant differences between different extract (*p* < 0.05).

Results of the DPPH radical scavenging activity, quantifying antioxidant activity of *Posidonia* leaf extracts, are displayed in Figure 2.

The lowest IC50 value (0.090 µg/µL), indicating the highest free radical scavenging ability, was observed in Gd-E4, comparable to the antioxidant power of the synthetic standards we utilized (Figure 2). On the contrary, as expected, the highest IC50 (14.530 µg/µL) was observed in brown leaf extract (B), not photosynthetically active leaves.

### 2.2. In Vitro Photo Protective Effect Evaluation of Posidonia Oceanica Extracts

**Figure 3:**
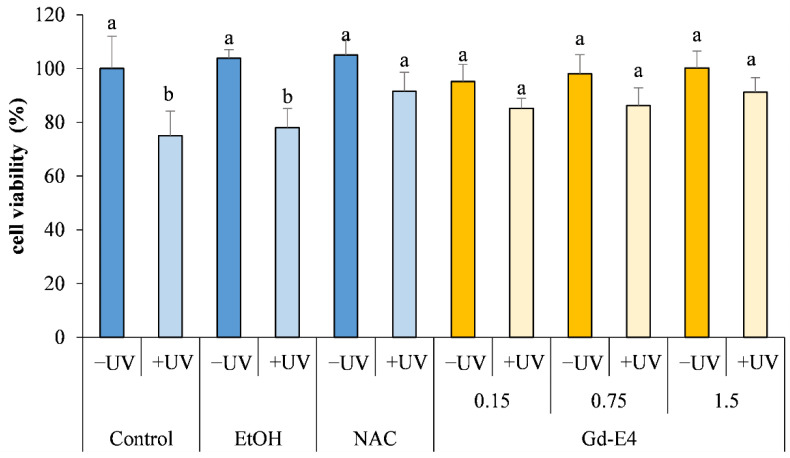
Cell viability test in HS-68 treatment with different concentration of Gd-E4 (ethanol 70% extracts from green leaves dried at 60 °C and ground four times) (0.15–1.5 µg/mL) for 24 h and subjected to the experiment induced stress with UV radiation. Control: fibroblasts maintained in standard culture conditions; EtOH: fibroblasts maintained in standard culture conditions with ethanol 0.1%; NAC: cell pretreated with the synthetic antioxidant N-acetylcysteine (5 mM); Gd-E4: cells pretreated with increasing doses of leaf extracts ground four times in ethanol 70%. −UV: fibroblasts not exposed to UV radiation; +UV: fibroblasts exposed to UV radiation. Bars represent the mean ± sd (*n* = 6). Different superscript letters indicate statistically significant differences (ANOVA; *p* < 0.05) among group.

On the basis of the preliminary evaluation of the antioxidant activity (Table 1), Gd-E4 extracts were selected to determine the photo protective capacity in vitro. Results showing responses of fibroblast not exposed to UV (-UV) to an increasing concentrations of Gd-E4, expressed as a percentage of viable cells compared to not extract treated cells (Control), are reported in Figure 3. Concentrations of Gd-E4 between 0.75–1.5 µg/mL did not induce any significant variation on the viability after 24 h, compared to control not exposed to UV (-UV) (Figure 3). 

Obtained MTT test results (Figure 3) indicate that UV exposure of fibroblast not treated with extracts (Control +UV) and fibroblasts treated with ethanol, the solvent used to vehicular the extract to the cultured cells (EtOH +UV), induced a significate toxicity (*p* < 0.05), expressed by decrease of viability. On the contrary, pre-treating cells with different concentrations of Gd-E4 prior to exposure to UV light resulted in a photo protective effect, as demonstrated by an increase in cell viability, comparable to cells pretreated with synthetic antioxidant NAC (Figure 3).

## 3. Discussion

The marine environment constitutes a generous source of biologically active compounds useful for human health and for this reason, attracting a growing scientific research attention. In the last 50 years, about 25,000 marine bioactive compounds have been identified, with an increase of 5%/year [36]. Seaweeds and seagrasses represent a potential source of unique molecules, characterized by strong biological activities; they are therefore of major importance to humans [2,3,4,5,6,7]. A peculiar characteristic of algae and marine plants is the production of secondary metabolites, synthesized by metabolic pathways that do not participate in primary functions, but are used for their adaptation to environmental stress conditions and as defense against pathogens and predators [3]. In particular, the polyphenol production, as defence strategy involving at least 23 bioactive compounds, has been reported in *P. oceanica* [1,3,20,22,37]. Antioxidant and pharmacological properties identified in seagrasses are mostly linked to the presence of phenolic compounds, in particular phenolic acids and flavonoids [9]. These compound can be reducing agents, donors of electrons or hydrogen atoms [38] and heavy metals binder [39]. 

In this study, the polyphenolic content and the antioxidant capacity were determined in extracts obtained from *P. oceanica* leaves, with different physiological stage, identified by leaves color (green, half-green and brown) (Figure 1). In fact, HPLC analysis (Table 1) revealed that the profile of some specific phenolic compounds showed significant differences according to the colors of the leaves. Extracts obtained from green leaves (G), rich in chlorophyll and photosynthetically active, contained more bioactive phenolic compounds than extracts obtained from half brown (H) and brown (B) leaves. We considered then green leaves more suitable for our experimentation. 

The effects of the grinding process before extraction was investigated. In fact, it has been described that smaller leaf particle size guarantee a better access of the extraction solvent and then a better yield [40].

In our study, extraction by different solvents of leaves ground once (coarse particles) or ground four times (fine particles) were compared. The extraction yield and antioxidant activity strongly depended on the polarity of the solvent, which influences both qualitatively and quantitatively the extracts [23,41]. The impact of the solvent used for extraction is well-documented, being mixture of methanol, ethanol or acetone with water the best solvents [42,43,44]. 

Ethanol extracts of fine particles of dried *Posidonia* leaves had a higher yield of total phenolic compounds (Table 1), dominated by chicoric acid, as already described by Grignon-Dubois and Rezzonico [45]. Gd-E4, besides having a higher polyphenolic content (19.712 ± 0.496 mg GAE/g), showed a higher antioxidant activity (Figure 2); in fact, Gd-E4 was the extract that has the lowest IC50 (0.090 µg/µL), comparable to that of synthetic antioxidants.

Drying by heat of the vegetal sample before extraction is a very complex stage. Some studies suggested that oven drying of the sample reduces drastically the yield and the bioactivity of the extracts [46,47,48]. High temperatures and oxygen expositions tend to damage the compounds of interest. However, on the other hand, some studies showed that the drying increased yields and bioactivities [44], or at least does not have any effect [49]. 

Drying temperature and time are factors that can influence the efficiency of the heat treatment. Phenolic compounds are thermo-sensitive so a high drying temperature can damage them [50]. But on the other hand, a long drying time means a long exposition to oxygen, which can also degrades polyphenols [50]. Leaves can be dried for a long time at low temperature or over a short time at a high temperature. To decide which one of these procedures was the most appropriate, we compared *Posidonia* leaf extracts dried over 5 days at 37 °C with extracts obtained from of leaves dried over 2 days at 60 °C. On the basis of obtained results, the best yield and bioactivity were obtained by extracts from leaves dried over 2 days at 60 °C. According to a Mrkìc et al. [51], the critical factor is indeed the drying time. A heat treatment over a short time at a high temperature seemed to be the best to recover phenolic compounds. Cannac et al. [49] also showed that water evaporated from leaves *P. oceanica* leaves very quickly because they were easily desiccated, because of their perpetual immersion. Thus, based on our results, for *P. oceanica*, we recommend rapid drying to preserve the compounds and maximize their recovery.

Cosmeceutics represent a promising area for valorization and use of marine bioactive molecules. Nowadays, plant-based cosmetics are expanding their popularity because of the significant impact these substances have on the prevention of skin aging [52]. Various molecules isolated from marine organisms have proven to be efficient against photo-induced oxidative damage to the skin [53]. 

Since secondary metabolites contribute to plant photoprotection [54], they are interesting candidates for the prevention of adverse effects caused by UV radiation on human skin cells [55]. In addition, they display important anti-aging potency, for example resveratrol has action on longevity factors [56,57]. A very interesting aspect is the antitumor effect provided by the ability of the extract of *P. oceanica* to reduce the expression and activity of gelatinase on human fibrosarcoma cells and human melanoma cell line, preventing cell invasion [20,58].

The Gd-E4 extract was selected to assess the protective extracts in vitro effect of *P. oceanica*, as it had both the highest polyphenolic content (Figure 1) and the highest antioxidant activity (Figure 2).

Results showed no significant adverse effects on viability of *Posidonia* leaf extracts (Figure 3). 

Subsequently, the possible protective effect of Gd-E4 extract was investigated following exposure to UV, a known source of oxidative stress [59,60].

Analysis conducted through the MTT assay, showed that exposure to UV is able to induce a significant change in terms of density and cellular morphology, in cells exposed to UV without any pre-treatment with antioxidants compared to those previously treated with the extract Gd-E4 (Figure 3).

The exposure of cells to UV led to a significant increase in ROS and that, in contrast, exposure of cells to the same conditions, but with a pre-treatment (Gd-E4), showed improvement of cells viability. 

Since it appears to be well documented the positive relationship between polyphenol content of plants extracts and their antioxidant activity in vitro [11,23,25,61,62,63,64], the high content of total phenolic compounds found in Gd-E4 extract, may explain its activity against oxidative stress.

Ultra-violet radiation (UVA and UVB) exposition, due to their capacity to generate free radicals, especially reactive oxygen species, in the cells and the establishment of a condition of oxidative stress, can lead to skin cancer and other photoaging complications [53,65]. The cytotoxic effect of UV responsible for damage to all biological macromolecules and cell viability, has been widely demonstrated [59,66]. Ultra-violet radiation has the ability to induce the apoptotic cascade event in animal cells [66] and human cells [36,67]. 

Numerous UV-absorbing compounds have been extracted and isolated from plants [67,68] and various marine organisms (algae, fungi, lichens, bacteria, phytoplankton, animals, and plants) for the development of anti-photoaging and photo protective natural products [53]. Phenolic compound extracted from *Posidonia* leaves could be considered as valuable candidates for the formulation of cosmetic products utilized for UV protection.

## 4. Materials and Methods

### 4.1. Samples Collection, Processing and Preparation of Plant Extract

*Posidonia oceanica* is a protected species by the European ’Habitats’ Directive 92/43/EEC [15], Barcelona Convention (1976, amended in 1995) and Annex 1 of the 1979 Bern Convention on the Conservation of European Wildlife and Natural Habitats-RS 0.455. Referring to the Italian legislation concerning the management of *P. oceanica* banquettes [17,18,19], fresh foliar bundles naturally detached from the meadow (e.g. due to sea storms) and not yet deposited along the coast, were collected in late winter and early spring (between February and March), before forming banks along the coast of Favignana, one of the islands of the Egadi Marine Protected Area.

A total of 10 kg of fresh detached foliar fascicles, not yet deposited along the coast to form banquettes, were collected monthly, which represent a negligible part of the annual casted leaves, considering that previous studies [69,70] reported the annual beaching of 3251 metric tons DW of *P. oceanica* along 18.2 km of coast in Sardinia and 72.2 metric tons DW along the Maltese coast in beaches extended between 0.35 and 112.8 km.

The sustainability of the collection method of *P. oceanica* foliar bundles, for the extraction of bioactive molecules, was validated by the Managing Authority of the Egadi Islands Marine Protected Area.

This method guaranteed the preservation of both the *P. oceanica* meadow, which remained untouched maintaining its important ecological role and of the banquettes that have an important role in the shore morph-dynamics and coastal stability [71], as the collecting took place before its formation.

Before each handling, the *P. oceanica* foliar bundles were selected and the basal sheath (or petiole) was removed and discarded to facilitate drying and extraction. 

**Figure 4:**
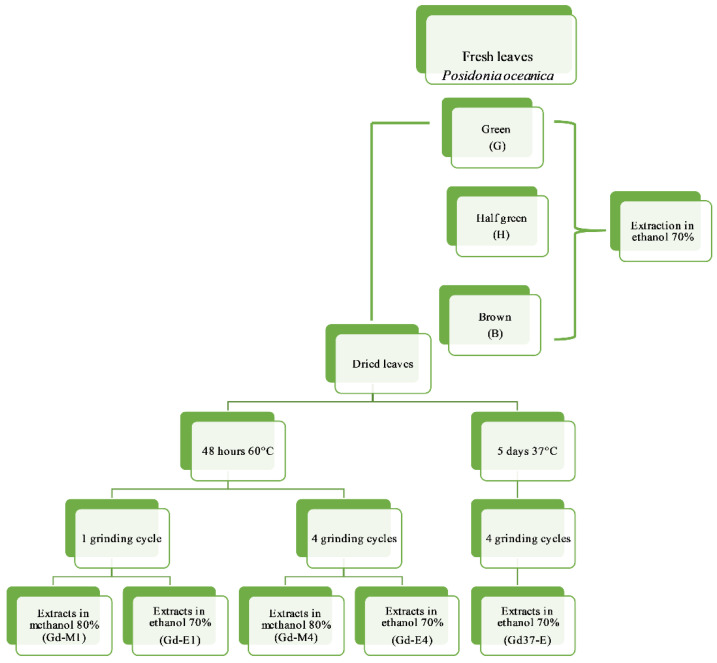
Flowchart of the experimental design.

The leaves of *P. oceanica* were processed as shown in Figure 4. 

The leaves of *P. oceanica* were extracted fresh and dried. Two procedures were carried out to dry the green leaves: in the first procedure the leaves were dried for 48 h at 60 °C, ground into fine powder [23], carrying out for part of the leaves one grinding cycle and the remaining part four grinding cycles; in the second procedure the leaves were dried for 5 days at 37 °C ground into fine powder with four grinding cycles.

#### Extraction

The leaves of collected bundles were divided into three batches, according to their photosynthetic capacity, determined by different colors: green leaves (G), half-green leaves, (H) and brown leaves (B), being the brown senescent leaves photosynthetically inactive. Five g of fresh green (G), half-green, (H) and brown leaves (B) were extracted by 10 mL of 70% ethanol homogenizing leaves for five minutes at 4 °C, using an Ultra-Turrax (IKA, Werke Staufen, Germany) at 24,000 rpm [72,73,74]. Green leaves were dried at 37 °C and 60 °C and submitted to one cycle or 4 cycle of grinding, lasting 10 min, by Grindomix GM200. One g of ground dried leaves was extracted by 10 mL of methanol 80% or ethanol 70%, Gd-M1 and Gd-E1, one grinding cycle and Gd-M4 and Gd-E4 four grinding cycles. Green leaves dried at 37 °C underwent 4 grinding cycles and were extracted with ethanol at 70% (Gd37-E).

The extracts were centrifuged (1100× *g* at 4 °C for 10 min) and the supernatant was recovered in a tube and stored at −20 °C.

The residue was solubilized and the extraction was performed with 10 mL of solvent as above. The recovered solvent was pooled with the previous extract. In the last extraction cycle the residue was again put back into solution with 10 mL of solvent and left to shake overnight at room temperature. The extract was centrifuged (1100× *g* at 4 °C for 10 min) and the supernatant was recovered and combined with the previous extract.

The contents of the extractions were transferred into a tube of known weight.

The extract was vacuum dried, weighed and finally re-suspended with a known volume of solvent to obtain a final concentration of 100 mg/mL or 50 mg/mL.

### 4.2. Characterization of the Antioxidant Power

#### 4.2.1. Total Polyphenols Contents

Total phenolic content of samples was determined by Folin-Ciocalteu reagent assay [75,76]. 

Gallic acid (Merck KGaA, Darmstadt, Germany) was used as standard for calibration (5–500 mg/mL) and results were expressed as mg of gallic acid equivalents (GAE) per g of the *P. oceanica* dw [23,25,26,77]. The absorbance of each dilution was measured at 725 nm, using a microplate reader (Multiskan-Sky Microplate Reader, Thermo-Scientific^TM^, Waltham, MA, USA). Each sample was analyzed in triplicate.

#### 4.2.2. HPLC Analysis of *Posidonia* Leaf Extracts

A known concentration of *Posidonia* leaf extract (10 mg/mL) was filtered on 0.2 µm filter (MiniUniprep™, Whatman, Maidstone, England) prior to injection and analyzed by RP-HPLC, utilizing an Elite LaChrom HPLC system equipped with HPLC pump L-7100 (LaChrom, Merck-Hitachi, Tokyo, Japan), Column oven L-7350 (LaChrom, Merck-Hitachi, Tokyo, Japan) and DAD detector L-2455 (Elite LaChrom, Merck-Hitachi, Tokyo, Japan). Data analysis was performed by EZChrom Elite V.3.1.7 software (Scientific Software, Pleasanton, CA, USA).

A column Kinetex C-18 (150 × 4.6 mm) ID 5 µm, 100 Å of shell size (Phenomenex, Torrance, CA, USA) was used at a constant temperature of 35 °C. The chromatographic separation of the extracts was obtained according to Puigventós et al. [78] with minor modifications. The binary gradient system was set with phase A (0.1% aqueous H_3_PO_4_, Merck, HPLC grade) and B (methanol, Chromanorm, VWR chemicals, HPLC grade), at a flow of 1.5 mL min^−1^, as follows: 0–3 min 5% B; 3–6 min from 5 to 25% B; 6–9 min from 25 to 37% B; 9–13 min 37% B; 13–18 min from 37 to 54% B; 18–22 min 54% B; 22–26 min from 54 to 95% B; 26–29 min 95% B; 29–29.15 min from 95 to 5% B; 29.15–36 min 5% B. Injection volume was 20 µL. Selected wavelengths for polyphenol analysis were 205, 280, 320 and 350 nm. HPLC grade (Merck KGaA, Darmstadt, Germany) Caffeic Acid, ρ-coumaric Acid, Gallic Acid, ρ-hydroxybenzoic Acid, Phloroglucinol, Quercetin, Trans-ferulic Acid and Vanillic Acid, were used as standards for identification and quantification of polyphenols in *Posidonia* leaf extracts according to Robbins and Bean [79]. Calibration curves were obtained using a broad range of standard concentration (0.0125 to 0.5 mg/mL) in methanol solution.

#### 4.2.3. DPPH Assay

DPPH Assay was performed reported by Brand-Williams et al. [80]. 

Samples were diluted in EtOH 96% at 10, 5 and 1 mg/mL and 20 µL of each dilution were added, in triplicate, to a 96 well plate. Standard curves were prepared for gallic acid, caffeic acid, ascorbic acid, alpha-tocopherol (Merck KGaA, Darmstadt, Germany), using six concentrations (10; 5; 1; 0.5; 0.1 and 0.05 mg/mL). A total of 200 µL of 60 mg/mL DPPH (Merck KGaA, Darmstadt, Germany) were added to each well (except the blank). Absorbance was read after 30 min. The percentage of DPPH inhibition was obtained by the following equation

I% = 1 − (A_sample_/A_blank_) × 100% (1)

The inhibition values were calculated for extracts at concentrations 1, 5 and 10 mg/mL and the slope of the linear portion of each graph was used to calculate IC 50% [23,81].

### 4.3. Evaluation of Bioactive Properties In Vitro

#### 4.3.1. Cell Culture 

Human skin fibroblast (HS-68) (Sigma-Aldrich, St. Louis, MO, USA) were grown as a monolayer in flasks, using Dulbecco’s Modified Eagle’s Medium (DMEM) supplemented with 10% fetal bovine serum (FBS), 2 mM glutamine, and 100 µg/mL penicillin–streptomycin, incubated in a humidified atmosphere at 5% CO_2_, 95% air and 37 °C, under sterile conditions. 

#### 4.3.2. Evaluation of Photo-Protective Effect in Fibroblast Cell Line HS-68

Cells at 80% of confluence were detached, utilizing a trypsin solution (0.05% of trypsin in PBS, pH 7.2–7.4) and pelleted by centrifugation (1000 rpm, 10 min, 25 °C). The cell suspension was dispensed in 96-well plate at a density of 8000 cells/well and incubated for 24 h before the exposure to ethanol extract of *P. oceanica* leaves (Gd-E4). 

The extracts (dissolved in ethanol) were diluted in culture medium at a concentration ranging from 0.15–1.5 µg/mL, at a final solvent concentration not exceeding 0.1% (*v/v*). As already assessed [25] this solvent concentration did not exerts any effects on cell viability. After 24 h, cells were incubated at increasing concentrations of Gd-E4 extract (0.15–0.75–1.5 µg/mL) and then UV-irradiated. N-acetylcysteine (NAC 5 mM), a synthetic antioxidant, that inhibits oxidative stress formation, was used as control [25,82]. 

To prevent UV light absorption by the cell culture medium, the medium was removed just prior to irradiation and replaced with a thin layer of PBS to cover the cells. All samples were exposed to UV radiation (lamp UV KW 254 nm) at dose rate of 105 erg/mm^2^/sec for 5 min, with a daily increase of 5 min, for a total of three days of exposure [59,83]. Cells not treated with Gd-E4 extract and not UV irradiated were cultivated as controls.

The viability of cells was measured using the MTT assay according to Mosmann [84]. Results were expressed as percentage of viable cells, respect to controls. Each experiment of viability was carried in six replicates.

### 4.4. Statistical Analysis

Data are reported as mean values ± standard deviation.

One-way analysis of variance (ANOVA) was performed and Tukey was used as post hoc test for comparison of the means among samples. The degree of heterogeneity was assessed by the Cochran test [85] Significance was accepted at probabilities *p* < 0.05. ANOVA performed using STATISTICA (version 8.0, Statsoft Inc., Tulsa, OK, USA).

## 5. Conclusions

In addition to its significant ecological role, thanks to bioactive properties of its compounds, *P. oceanica* could be considered as a promising resource for human health.

This study provides a demonstration of the possible use of natural extracts obtained from fresh beach cast *P. oceanica* leaves as antioxidant and photo-protective agents for the skin protection. 

Human skin was the main photo-oxidative target, being continuously exposed to UV radiation. This exposure can trigger a state of oxidative stress and damage at the cellular level. Scientific research has focused on the discovery of new natural compounds of marine origin in order to obtain molecules with important therapeutic properties for human health. Polyphenolic extracts from marine plants are potential candidates for the prevention of the harmful effects of UV radiation on the skin.

From our results, it can be concluded that the intrinsic antioxidant capacity of *P. oceanica* leaves extract ensures increased antioxidant capacity at the cellular level, confirming the importance of using in vitro cell systems and biomolecular markers for the research of bioactives for cosmeceutical applications. This allows reliable results to be obtained in order to validate the efficacy of bioactive molecules of marine origin. 

This work contributes to the improvement of current research strategies thus allowing the development of future perspectives to better understand the mechanisms of photo-acclimation in marine flora and fauna and will enable us to obtain potent compounds that are beneficial for cosmeceutical and pharmaceutical applications.

## Figures and Tables

**Table 1:** Concentrations (µg/g) in phenolic compounds of extracts of leaves of *P. oceanica* with different treatments. G: green fresh leaf ethanol 70% extracts; H: half-green fresh leaf ethanol 70% extracts; B: brown fresh leaf ethanol 70% extracts; Gd-M1: methanol 80% extracts from green leaves dried at 60 °C and ground once; Gd-M4: methanol 80% extracts from green leaves dried at 60 °C and ground four times; Gd-E1: ethanol 70% extracts from green leaves dried at 60 °C and ground once; Gd-E4: ethanol 70% extracts from green leaves dried at 60 °C and ground four times; Gd37-E: ethanol 70% extracts from green leaves dried at 37 °C and ground four times.

Phenolic Compounds	G	H	B	Gd-M1	Gd-M4	Gd-E1	Gd-E4	Gd37-E
Phloroglucinol	25.606	4.131	15.831	95.95	104.603	82.159	102.085	117.296
Gallic Acid	103.159	25.572	29.57	299.883	271.192	199.412	411.845	472.836
ρ-hydroxybenzoic Acid	208.729	6.675	7.91	0	0	2.714	519.272	110.807
Vanillic Acid	76.269	3.857	21.942	111.614	78.035	66.206	131.375	161.822
Caffeic Acid	36.889	5.012	12.869	153.203	128.947	87.069	193.761	120.565
ρ-coumaric Acid	25.237	6.749	13.033	92.729	39.48	35.685	87.501	150.997
Ferulic Acid	23.335	9.32	15.635	86.098	60.814	46.106	83.422	82.53
Chicoric Acid	48.746	4.385	12.704	1873.188	2270.211	1208.203	4991.813	2843.772
Quercetin	26.993	1.992	9.71	65.891	57.743	46.599	105.185	135.457
Total	574.963	67.693	139.204	2778.556	3011.025	1774.153	6626.259	4196.08
